# An InN/InGaN Quantum Dot Electrochemical Biosensor for Clinical Diagnosis

**DOI:** 10.3390/s131013917

**Published:** 2013-10-15

**Authors:** Naveed ul Hassan Alvi, Victor J. Gómez, Paul E.D. Soto Rodriguez, Praveen Kumar, Saima Zaman, Magnus Willander, Richard Nötzel

**Affiliations:** 1 ISOM Institute for Systems based on Optoelectronics and Microtechnology, ETSI Telecomunicación, Universidad Politécnica de Madrid, Ciudad Universitaria s/n, Madrid 28040, Spain; E-Mails: vjgomez@isom.upm.es (V.J.G.); p.soto@isom.upm.es (P.E.D.S.R.); praveen.kumar@isom.upm.es (P.K.); 2 Department of Science and Technology (ITN), Campus Norrköping, Linköping University, Norrköping 60174, Sweden; E-Mails: saiza@itn.liu.se (S.Z.); magwi@itn.liu.se (M.W.)

**Keywords:** functionalized InN quantum dots, highly sensitive and efficient biosensors, potentiometric, clinical diagnosis

## Abstract

Low-dimensional InN/InGaN quantum dots (QDs) are demonstrated for realizing highly sensitive and efficient potentiometric biosensors owing to their unique electronic properties. The InN QDs are biochemically functionalized. The fabricated biosensor exhibits high sensitivity of 97 mV/decade with fast output response within two seconds for the detection of cholesterol in the logarithmic concentration range of 1 × 10^−6^ M to 1 × 10^−3^ M. The selectivity and reusability of the biosensor are excellent and it shows negligible response to common interferents such as uric acid and ascorbic acid. We also compare the biosensing properties of the InN QDs with those of an InN thin film having the same surface properties, *i.e.*, high density of surface donor states, but different morphology and electronic properties. The sensitivity of the InN QDs-based biosensor is twice that of the InN thin film-based biosensor, the EMF is three times larger, and the response time is five times shorter. A bare InGaN layer does not produce a stable response. Hence, the superior biosensing properties of the InN QDs are governed by their unique surface properties together with the zero-dimensional electronic properties. Altogether, the InN QDs-based biosensor reveals great potential for clinical diagnosis applications.

## 1. Introduction

The development of highly sensitive and fast biosensors with good reproducibility and specificity is the main focus of the biosensing research community because it offers a great opportunity for the diagnosis of many major life threatening diseases and their treatments at early stages. Therefore, the health industry urgently needs the development of more efficient, reliable, and cheap sensing and detection technologies. Towards this goal we demonstrate epitaxially grown InN/InGaN quantum dots (QDs) for highly sensitive, fast, and efficient potentiometric biosensors owing to their low-dimensionality and unique electronic properties. The excellent semiconducting properties of Group III-V compounds have led to many applications in electronic and optoelectronic devices. Among Group III-V semiconductor materials, InN has attracted a lot of attention for the development of electronic and photonic devices based on the recent progress in the synthesis of InN based novel nanostructures [1–5].

It is now established that unintentionally doped InN nanostructures contain an intrinsic electron accumulation layer at the surface due to a high density of surface donor states, which is very unusual among Group III-V semiconductors. It has been reported that the density of the positively charged surface donor states of InN nanostructures is as high as 10^13^ cm^−2^, which represents the highest electron accumulation observed in Group III-V semiconductor nanostructures [6–11].

The biological applications of InN nanostructures are largely unexplored. InN has only recently been considered as an appealing candidate for the detection of biological molecules and chemical species due to its unique properties, such as the high electron concentration near the surface, high chemical stability, and high sensitivity to charges in the environment [12–17]. Based on these distinct and excellent properties, we propose to develop an efficient InN QDs-based biosensor for medical diagnosis and demonstrate it for the capability of detecting changes in the concentration of cholesterol in the human body in real time.

The development of a biosensor based on epitaxial InN QDs taking additional advantage of their low dimensionality, high surface to volume ratio, and the planar arrangement, promising strong and rapid signal response is potentially very interesting. The high density of positively charged surface donor states of the InN QDs should facilitate electron transfer from the molecule to be detected, *i.e.*, oxidized [18] to the InN QD surface. This is the base of the sensor principle to determine, in the present case, the cholesterol concentration by measuring the potential difference which is established by the electron transfer between the electrically contacted InN QDs and a Ag/AgCl reference electrode.

Among the many techniques for the sensing of bio-molecules the enzymatic electrochemical biosensing technique is an important alternative to other non-enzymatic biosensing techniques as it requires only a simple experimental setup, short data acquisition time, and inexpensive chemicals. The enzymatic biosensors are fabricated by immobilization of enzymes onto the surface of the biocompatible sensing material which are selected according to the target molecules. Cholesterol oxidase (ChOx) is the most commonly employed enzyme in cholesterol biosensors because of its high selectivity to cholesterol molecules.

Here we present a biosensor based on InN QDs functionalized through immobilization of cholesterol oxidase onto the surface. The fabricated biosensor exhibits a fast and stable output response along with good linear sensitivity over a wide logarithmic cholesterol concentration range from 1 × 10^−6^ M to 1 × 10^−3^ M. Additionally, the presented biosensor reveals a rapid response time and good selectivity and repeatability at room temperature. It also exhibits good reusability over extended periods of time. We also compare the biosensing properties of the InN QDs with those of an InN thin film having the same surface properties, but different morphology and electronic properties. The sensitivity of the InN QDs based biosensor is twice that of the InN thin film based biosensor, the EMF response is three times larger, and the response time is five times shorter. The response in the case of a bare InGaN layer is not stable. This reveals that the superior biosensing properties of the InN QDs, which have been previously also observed for the detection of glucose [19] are governed by their unique surface properties together with the zero-dimensional nature.

## 2. Device Fabrication and Experimental Methods

The chemicals used were ChOx with the concentration of 25 U/mg from Sigma Aldrich (Stockholm, Sweden). The ChOx stock solution was prepared by dilution with tris–HCl (1 × 10^−2^ M). Cholesterol powder (Sigma), Triton X-100 (Sigma Aldrich), and phosphate buffered saline (PBS) tablets (Medicago, Québec, Canada) were used for preparation of the nutrient cholesterol solutions with different concentrations ranging from 1 × 10^−6^ M to 1 × 10^−3^ M. As cholesterol powder is insoluble in water, a PBS solution which contained 1% of Triton X-100 was added as solvent.

For biosensor fabrication, the immobilization of the cholesterol oxidase (ChOx) enzyme was carried out electrostatically by dipping the InN QDs, InN thin film, and InGaN layer samples into 50 μL of enzyme solution for 20 min and then drying in air for 2 h. To reduce the possible leaching of the enzyme and to eliminate foreign interferences a 5 μL aqueous solution of 2.5% glutaraldehyde and 0.5% Nafion was applied onto the electrode surface. All cholesterol biosensors were stored at 4 °C in dry condition when not in use.

The cholesterol solution was prepared by dissolving 500 mg of standard cholesterol in isopropanol, Triton X-100 and phosphate buffer (pH of 7.0) by stirring at 65 °C until the solution was clear and colorless. The isopropanol, Triton X-100, phosphate buffer ratio was 10:4:86 by weight. The potentiometric measurements were performed using the functionalized InN QDs, InN thin film, and InGaN layer samples as working electrode and a Ag/AgCl reference electrode from Metrohm Nordic (3MKCl, Bromma, Sweden), and recorded by a computer controlled Keithley 2400 source meter (Keithley Instruments, Inc., Cleveland, OH, USA).

## 3. Results and Discussion

**Figure 1.**
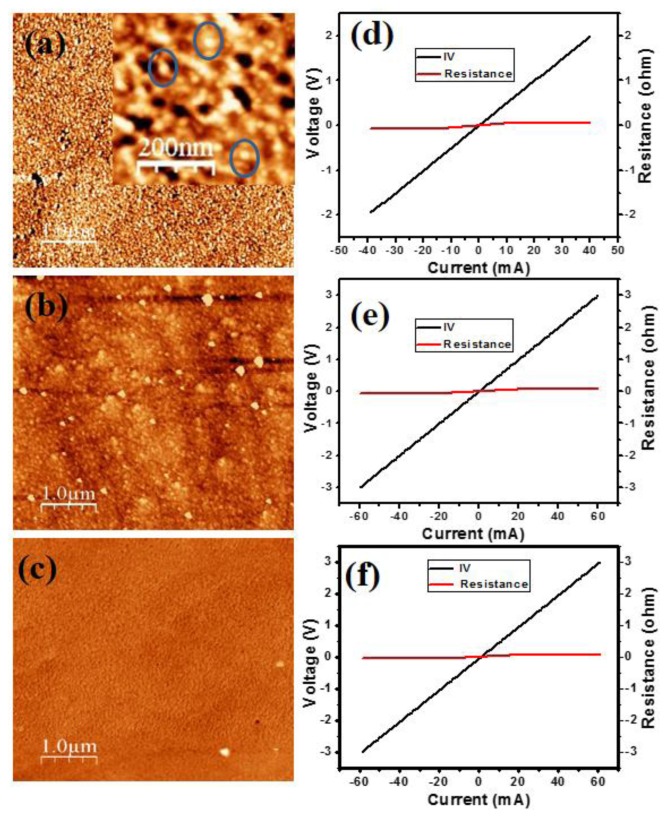
(**a**) AFM image of the InN QDs grown on an InGaN layer. Inset: AFM image with enlarged magnification. Some InN QDs are encircled for clarification; (**b**) AFM image of the InN thin film grown on an InGaN layer; (**c**) AFM image of the bare InGaN layer; (**d**–**f**) I-V curves measured with two Al ohmic contacts deposited on the InN QDs, InN thin film, and InGaN layer, respectively.

Figure 1a shows the atomic force microscopy (AFM) image of the two monolayer InN QDs grown on a 80 nm thick In_0.54_Ga_0.46_N layer on a (0001) GaN/sapphire substrate by plasma assisted molecular beam epitaxy (PA-MBE). In the inset the magnification is enlarged. Figure 1b shows the AFM image of the three monolayers InN thin film, and the AFM image of the bare InGaN layer is shown in Figure 1c. The growth was performed at 460 °C under slightly N-rich conditions. Details of the growth and characterization, in particular photoluminescence measurements, are presented in [20].

The QDs exhibit a height of 2–3 nm, diameter of 20–30 nm, and density of 4.5 × 10^10^ cm^−2^. The InN thin film and InGaN layer have a smoothly modulated surface. Figures 1d–f depict the I-V curves measured with two Al contacts, half a millimeter apart, deposited on the InN QDs, InN thin film, and bare InGaN layer, respectively, which show excellent ohmic behavior with low resistance. This is due to the high n-type conductivity commonly established in high-In-composition (>50%) InGaN layers due to native defects.

**Figure 2.**
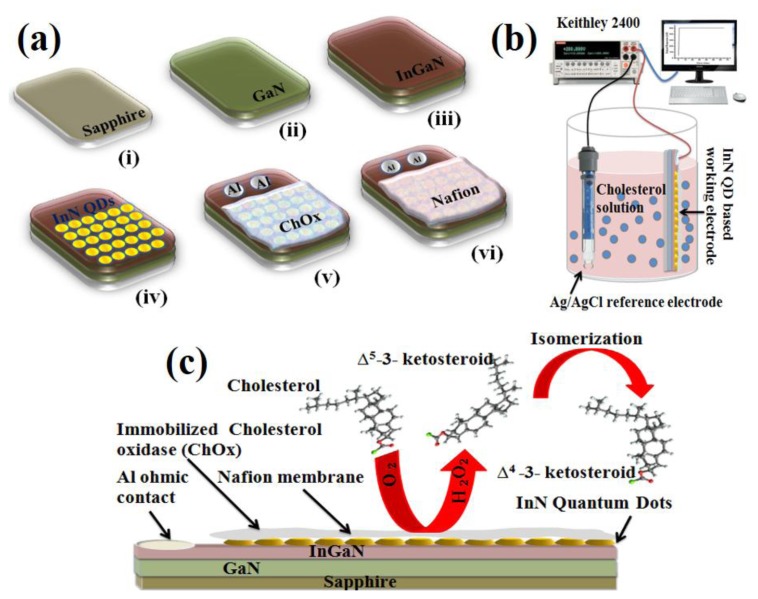
(**a**) Schematic diagram of the fabrication process of the biosensor; (**b**) Schematic illustration of the sensing setup using the working electrode comprised of the InN QDs coated with ChOx and a Ag/AgCl reference electrode; (**c**) Schematic illustration of the working electrode comprised of the InN QDs coated with ChOx along with the possible electrochemical reaction near the electrode.

Figure 2a shows the schematic diagram of the fabrication process of the cholesterol biosensor, Figure 2b the schematic illustration of the cholesterol sensing setup, and Figure 2c the schematic illustration of the cholesterol working electrode comprised of the functionalized InN QDs. It also illustrates the electrochemical reaction near the biosensing electrode.

The working mechanism of the cholesterol biosensor is explained by taking into account the bi-functional role of ChOx, which not only provides good specificity for steroid attachment but also works as a catalyst to initiate the chemical reaction. The enzyme catalytic reaction between cholesterol molecules and oxygen in the electrolyte solution produces Δ^5^-3-ketosteroid and hydrogen peroxide as shown below [21,22]:

(1) $$\text{Cholesterol}+{\text{O}}_{2}\overset{\text{ChOx}}{\to }\Delta^{5}‐3‐\text{Ketosteroid}+{\text{H}}_{2}O_{2}$$

However, the unstable nature of the Δ^5^-3-ketosteroid leads to immediate isomerization to produce a stable Δ^4^-3-ketosteroid as the result of an intra-molecular shift of protons from the 4β to the 6β position:

(2) $$\Delta^{5}‐3‐\text{ketosteroid}\overset{\text{Isomerization}}{\to }\Delta^{4}‐3‐\text{ketosteroid}$$

This electrochemical reaction is responsible for the charge transfer near the surface of the working—and reference electrodes to produce the electrochemical cell voltage. In our process the enzyme activity is 0.35 UmL^−1^, where one U is the enzyme activity which oxidizes one μM cholesterol per minute under the essay at room temperature and pH of 7.

**Figure 3.**
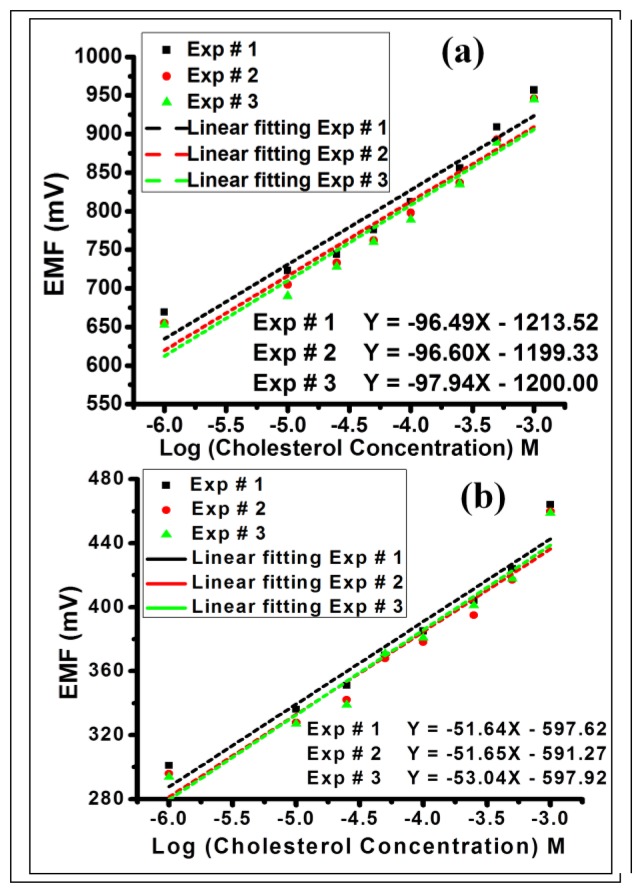
(**a**,**b**) EMF as a function of the logarithmic cholesterol concentration of the InN QDs and InN thin film based biosensors, respectively. Exp # 1–3 denote three different experiments.

Figure 3a,b depict the electrochemical cell voltage (EMF) response of the fabricated InN QDs and InN thin film based biosensors, respectively, measured for different cholesterol solutions with concentrations ranging from 1 × 10^−6^ M to 1 × 10^−3^ M. The EMF is linear *versus* the logarithmic concentration of cholesterol for both sensors. The EMF response of the InN QDs-based biosensor is increasing from 669 mV for 1 μM to 957 mV for 1 mM and it shows a significantly high slope of 96 mV/decade. The EMF response of the InN thin film-based biosensor is increasing from 301 mV for 1 μM to 464 mV for 1 mM, showing a slope of 51 mV/decade. The performance of the sensors is found to be independent of the sensing area that is in contact with the cholesterol solution and the quantity of the cholesterol solution. Repeated experiments (denoted Exp # 1–3) with the same biosensors show reproducible results, confirming the stability, linearity, and reusability of the fabricated biosensors.

**Figure 4.**
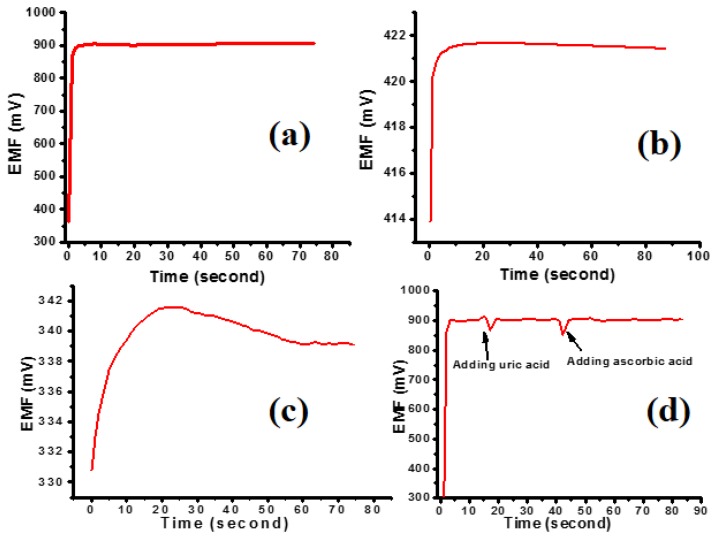
(**a**) EMF as a function of time of the InN QDs based biosensor for 500 μM cholesterol concentration; (**b**) EMF as a function of time of the InN thin film based biosensor for 500 μM cholesterol concentration; (**c**) EMF as a function of time of the InGaN layer based biosensor for 500 μM cholesterol concentration; (**d**) EMF as a function of time when adding 50 μM uric acid (UA) and ascorbic acid (AA) to the 500 μM cholesterol solution.

In Figure 4a,b the EMF response as a function of time of the InN QDs- and InN thin film-based biosensors is depicted. The biosensor based on the InN QDs delivers a five times faster output voltage (EMF) response in comparison with the biosensor based on the InN thin film. When dipping the InN QDs based biosensor into the cholesterol solution, the output signal is stable within 0.5% after two seconds (±0.5 s), while it takes about ten seconds for the InN thin film based biosensor. The time response of the InN QDs and InN thin film based biosensors is shown here for a 500 μM cholesterol solution. The EMF as a function of time, *i.e.*, the stability and time response are the same for higher and lower cholesterol concentrations are, therefore, independent of the concentration. The output voltage for the bare InGaN layer is significantly lower, as shown in Figure 4c, and it is not stable in time.

In the following we suggest an explanation of the superior sensing properties of the InN QDs with respect to the InN thin film which is based on the zero-dimensional electronic properties of the QDs together with the high density of positively charged surface donor states. For a density of positively charged surface donor states of the order of 10^13^ cm^−2^, about 40–70 donors are situated on the QDs when taking into account the QD diameter of 20–30 nm. Due to the zero-dimensional quantum confinement of carriers, however, the QDs each can accommodate only two electrons in the ground state. Even when considering the presence of excited states, this results in a local net positive charge of the QDs with the compensating electrons expelled to their surroundings. This positive net charge actively promotes the oxidation of cholesterol, *i.e.*, the transfer of electrons to the QD working electrode, setting the electrochemical potential with respect to the reference electrode. Notably, the experimental results show, that this can lead to a sensitivity which is beyond the Nernst limit of 59 mV/decade. For the InN thin film, on the other hand, the positively charged surface donors are uniformly compensated by the accumulated electrons in the semiconductor and no specific sites promoting the oxidation of cholesterol are present. For both structures, however, the overall high surface charge density leads to a stable response which is not given for the bare InGaN layer. A more quantitative analysis, including the detailed electronic structure of the InN QDs, InN thin film, and InGaN layer and the related electrochemical processes is far beyond the scope of the present study.

The selectivity with regard to well-known interfering agents such as uric acid and ascorbic acid of the InN QDs based biosensor is also studied. Upon adding 50 μM uric acid or ascorbic acid to the 500 μM cholesterol solution the output (EMF) signal does not substantially change. This is shown in Figure 4d illustrating the EMF as a function of time upon adding uric acid or ascorbic acid, indicated by arrows. This reveals that the presented biosensor has good selectivity which is attributed to the perm selective (charge-exclusion) property [23,24] of the Nafion film coated on the sensing electrode.

**Figure 5.**
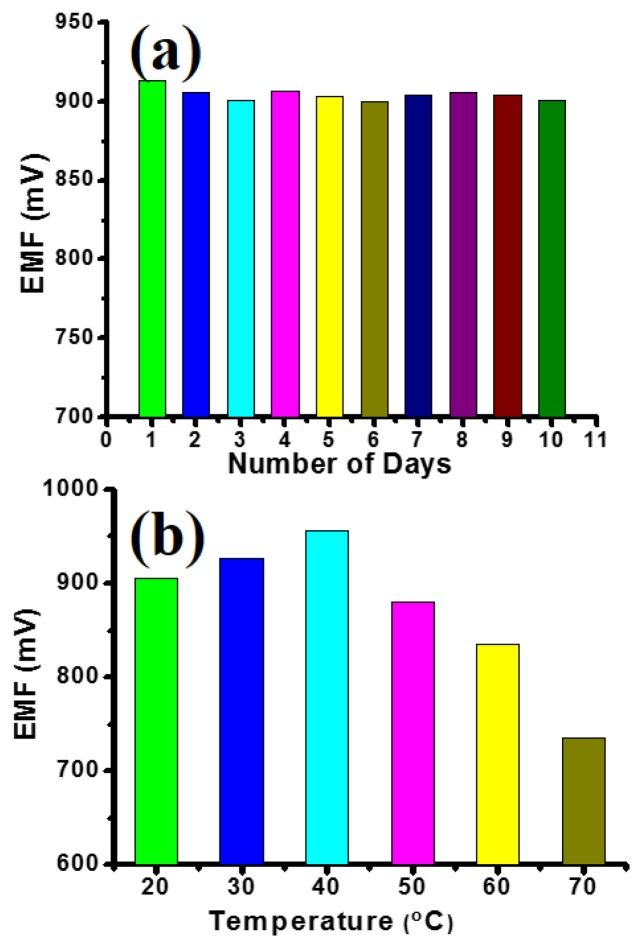
(**a**) Repeated experiments for ten consecutive days for 500 μM cholesterol concentration using same InN QDs-based biosensor; (**b**) EMF as a function of temperature of the InN QDs-based biosensor for 500 μM cholesterol concentration.

Moreover, the InN QDs-based biosensor exhibits excellent storage stability as evidenced by a series of repeated experiments for ten consecutive days. Figure 5a shows the EMF for 500 μM cholesterol concentration using same InN QDs-based biosensor. The EMF varies only marginally day-by-day and shows no degradation trend. These measurements are performed to ensure that the biosensor can be used for routine diagnosis retaining its sensitivity and reusability for long durations of time.

Figure 5b shows the effect of temperature on the output EMF response of the InN QDs-based biosensor. The output EMF response for a certain cholesterol concentration increases with increasing temperature up to 40 °C and then decreases. This reveals that the activity of the enzyme decreases at temperatures below 40 °C, as well as at temperatures above 40 °C leading to a decrease of the output EMF. Hence, the temperature has to be carefully controlled during cholesterol sensing measurements. This, however, is not related to the intrinsic sensing properties of the InN QDs, but rather their surface functionalization.

Notably, our InN QDs based biosensor exhibits excellent performance such as high sensitivity, rapid response time, good selectivity, and excellent reusability, comparable and in many aspects superior to other available enzymatic cholesterol biosensors, as shown in Table 1, which lists the functional properties of various enzymatic cholesterol biosensors developed using different sensing materials and detection techniques [25–31].

## 4. Conclusions

To summarize, we have fabricated a potentiometric biosensor for the detection of cholesterol based on bio-chemically functionalized InN QDs. The presented biosensor utilizes the substantial advantages of high surface charge density and low-dimensionality of the InN QDs to generate a highly sensitive and rapid response. We have investigated the sensitivity, selectivity, and reusability over a large cholesterol concentration range from 1 × 10^−6^–1 × 10^−3^ M. The biosensor had an excellent sensitivity slope of 97 mV/decade with a fast output response of two seconds. We have also compared the potentiometric biosensing properties of the InN QDs with those of an InN thin film. The sensitivity of the InN QDs-based biosensor was twice that of the InN thin film-based biosensor, the EMF was three times larger, and the response time was five times shorter. The response for a bare InGaN layer was not stable. Therefore, the superior biosensing properties of the InN QDs may be attributed to their unique surface properties, *i.e.*, high density of positively charged surface donor states, together with the zero-dimensional electronic properties. The high sensitivity, rapid response time, good selectivity, and excellent reusability reveal that our biosensor has great potential to be used for clinical diagnosis.

## Figures and Tables

**Table 1.** Functional properties of different available enzymatic cholesterol biosensors.

**Electrode Matrix**	**Detection Techniques**	**Sensitivity/Detection Limit**	**Response Time(s)**	**Reference**
ZnO nanorods	Potentiometric	35.2 mV/decade/0.001–10 mM	10	[25]
Tetraethylorthosilcate	Amperometric	2–12 mM	15	[26]
Controlled pore glass	Thermometric	0.1 mM	120	[27]
BSA/polycarbonate/oxygen electrode	Polarographic	0.1–2.75 mM (2–50 gm/dl)	∼90	[28]
ZnO nanowalls	Potentiometric	53 mV/decade/10^−6^–10^−3^ M	5	[29]
Polyaniline/ChOx	Spectrophotometric	3.016 (Abs·M^−1^·cm^−2^)/643 μM	-	[30]
Polyaniline-MWCNT/ChOx	Amperometric and spectrophotometric	6.8 (μA·mM^−1^·cm^−2^)		[31]
